# Membrane permeabilization of mammalian cells using bursts of high magnetic field pulses

**DOI:** 10.7717/peerj.3267

**Published:** 2017-04-26

**Authors:** Vitalij Novickij, Janja Dermol, Audrius Grainys, Matej Kranjc, Damijan Miklavčič

**Affiliations:** 1 Institute of High Magnetic Fields, Vilnius Gediminas Technical University, Vilnius, Lithuania; 2 Faculty of Electrical Engineering, University of Ljubljana, Ljubljana, Slovenia

**Keywords:** Electroporation, Electropermeabilization, Magnetopermeabilization, CHO cells, Propidium iodide, YO-PRO-1

## Abstract

**Background:**

Cell membrane permeabilization by pulsed electromagnetic fields (PEMF) is a novel contactless method which results in effects similar to conventional electroporation. The non-invasiveness of the methodology, independence from the biological object homogeneity and electrical conductance introduce high flexibility and potential applicability of the PEMF in biomedicine, food processing, and biotechnology. The inferior effectiveness of the PEMF permeabilization compared to standard electroporation and the lack of clear description of the induced transmembrane transport are currently of major concern.

**Methods:**

The PEMF permeabilization experiments have been performed using a 5.5 T, 1.2 J pulse generator with a multilayer inductor as an applicator. We investigated the feasibility to increase membrane permeability of Chinese Hamster Ovary (CHO) cells using short microsecond (15 µs) pulse bursts (100 or 200 pulses) at low frequency (1 Hz) and high dB/dt (>10^6^ T/s). The effectiveness of the treatment was evaluated by fluorescence microscopy and flow cytometry using two different fluorescent dyes: propidium iodide (PI) and YO-PRO®-1 (YP). The results were compared to conventional electroporation (single pulse, 1.2 kV/cm, 100 µs), i.e., positive control.

**Results:**

The proposed PEMF protocols (both for 100 and 200 pulses) resulted in increased number of permeable cells (70 ± 11% for PI and 67 ± 9% for YP). Both cell permeabilization assays also showed a significant (8 ± 2% for PI and 35 ± 14% for YP) increase in fluorescence intensity indicating membrane permeabilization. The survival was not affected.

**Discussion:**

The obtained results demonstrate the potential of PEMF as a contactless treatment for achieving reversible permeabilization of biological cells. Similar to electroporation, the PEMF permeabilization efficacy is influenced by pulse parameters in a dose-dependent manner.

## Introduction

Electroporation is a method of non-thermal treatment of biological cells or tissues by pulsed electric field (PEF) which results in transient membrane permeability increase and thus conditioning of molecular transmembrane transport mechanisms (Neumann & Rosenheck, 1972; Weaver, 2000; Kotnik et al., 2012; Rems & Miklavčič, 2016; Wagstaff et al., 2016). Flexible control of the cell membrane permeability to impermeable molecules by means of electroporation offers a vast array of applications, including, but not limited to, biotechnology, food processing and medicine (Mahnič-Kalamiza, Vorobiev & Miklavčič, 2014; Yarmush et al., 2014; Kotnik et al., 2015; Golberg et al., 2016b). Current state of the art of the biomedical electroporation involves application of various electrode array configurations (Campana et al., 2016b; Ongaro et al., 2016; Szczurkowska et al., 2016) for both invasive (i.e., tissue ablation Reberšek et al., 2014; Golberg et al., 2016a; Klein et al., 2016) and non-invasive (i.e., transdermal) electroporation applications (Blagus et al., 2013; Zorec et al., 2013; Becker et al., 2014; Chen et al., 2015). However, these applications require direct contact between the applicator, electrodes and the biological sample. Therefore, despite many applications, the methodology has considerable limitations such as the dependence of PEF distribution on the dielectric properties of the sample (Corovic et al., 2013; Peyman et al., 2015; Campana et al., 2016a; Liu et al., 2016), presence of electrochemical reactions in the electrode-electrolyte or tissue interfaces (Pataro et al., 2015) and the possibility of electrical breakdown between the electrodes (Guenther et al., 2015; Rubinsky et al., 2016).

New electroporation protocols are constantly being developed since electroporation is very sensitive to the homogeneity of the treated sample—e.g., the conductive gel in the electrode-tissue interface or the medium viscosity can alter treatment efficacy significantly (Ivorra et al., 2008; Sungailaite et al., 2015; Suzuki, Marques & Rangel, 2016). The non-homogeneous sample conductivity could be addressed by applying, high-frequency bipolar pulse protocols (H-FIRE) which mitigate the impedance differences (Bhonsle et al., 2015; Sano et al., 2015) but unfortunately require delivery of higher energy than longer unipolar irreversible electroporation (IRE) pulses (Murovec et al., 2016; Sweeney et al., 2016). However, universal protocols, which allow countering or optimising all the limitations described above, are still not available (Pakhomova et al., 2011; Blumrosen et al., 2016; Yildirim et al., 2016).

Recently, in addition to electroporation by PEF, the permeabilization method by pulsed electromagnetic fields (PEMF) has been proposed—the PEMF induced increase of the cell membrane permeability is similar to electroporation. However the effect does not depend on the conductance or the uniformity of the sample and at the same time is contactless (Grainys et al., 2012; Kardos & Rabussay, 2012; Towhidi et al., 2012; Shankayi, Firoozabadi & Mansurian, 2013; Kranjc et al., 2016). The proof of concept has been confirmed both *in vitro* (Towhidi et al., 2012; Shankayi et al., 2014; Novickij et al., 2015) and *in vivo* for the PEMF mediated transport of small molecules (e.g., cisplatin, platinum) (Kranjc et al., 2016) and large molecules (e.g., DNA) (Kardos & Rabussay, 2012). Currently however, the efficacy of the PEMF treatment is inferior to electroporation. Also, the permeabilization phenomenon by PEMF is not yet well understood. Namely, the electric field that is induced by the time-varying magnetic field is lower by several orders of magnitude than the electric field causing membrane permeabilization in conventional electroporation experiments (Lucinskis et al., 2014; Kranjc et al., 2016). The permeabilization effect is likely to occur during multiple high dB/dt, where dB/dt denotes the time change of the magnetic flux density (*B*) (Van Bree et al., 2013; Novickij et al., 2015), or during application of long train of microsecond pulses (Towhidi et al., 2012; Shankayi et al., 2014; Kranjc et al., 2016).

The non-straightforward dependence of the PEMF permeabilization on the treatment parameters requires further research of different protocols. Improvement of pulse generation, systematic research and parametric analysis may result in a novel contactless method that could be an attractive alternative to the conventional electroporation. Previously, it was shown that the repetitive (40 Hz) high dB/dt (8 × 10^5^ T/s) magnetic fields up to 3 T induce permeabilization of only a small fraction of the cells (Novickij et al., 2015). However, Towhidi et al. (2012) have shown that the application of lower frequency pulses increases the treatment efficacy. Therefore, in this work we have redeveloped and improved the pulsed magnetic field setup by doubling the accumulated pulse energy which allowed achieving maximum pulse amplitude of 5.5 T. We have investigated if the low frequency (1 Hz), but high dB/dt pulsing protocol will allow achieving high permeabilization of Chinese Hamster Ovary (CHO) cells determined by the cell membrane permeabilization and survival assays.

## Materials & Methods

### Pulsed power setups

**Figure 1:**
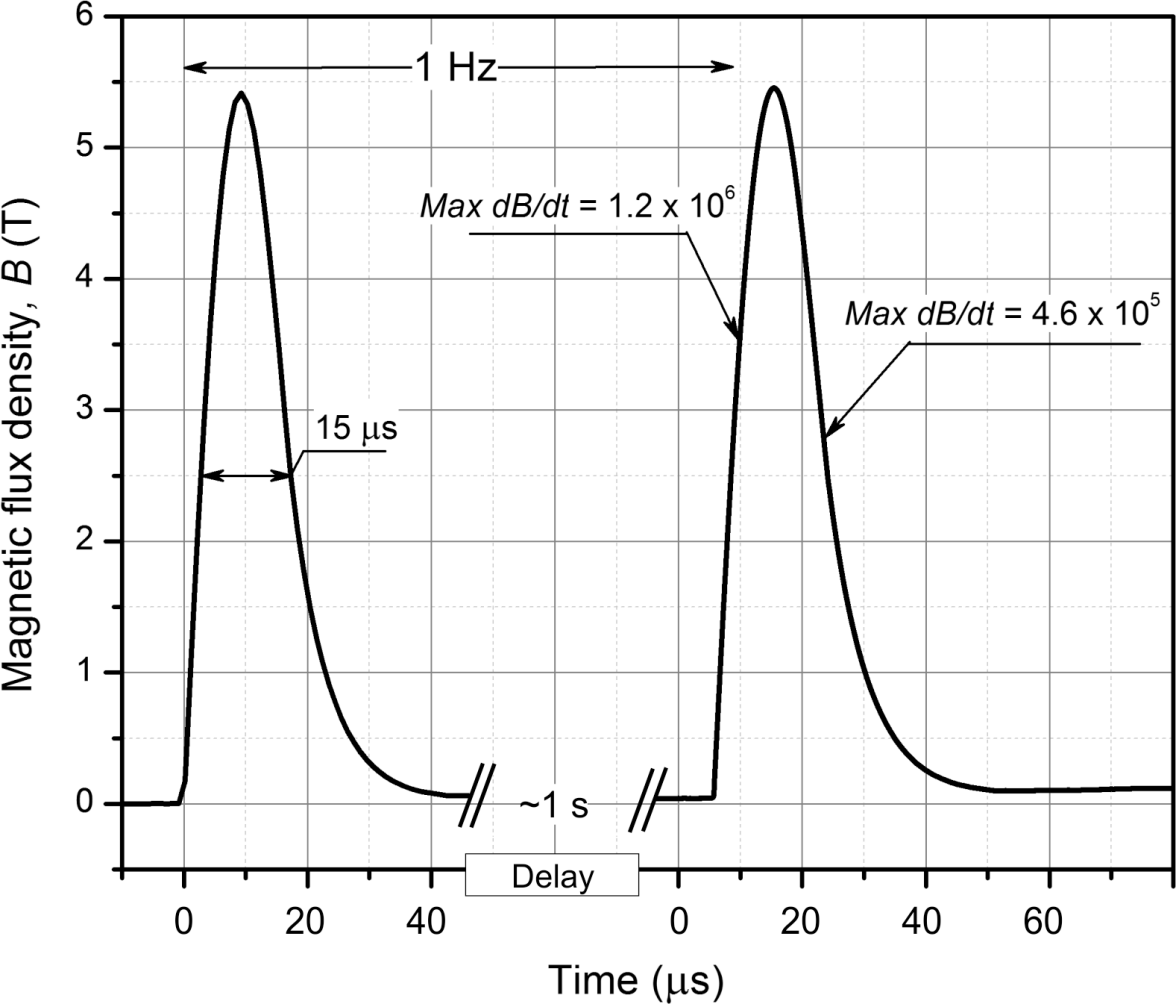
The waveform of the high dB/dt magnetic field pulse. Each magnetic pulse was 15 µs wide and had a peak of 5.5 T (Acquired using Tektronix DPO4034, post-processed in OriginLab 8.5).

For the pulsed magnetic field generation, the 550 A up to 2 kV pulsed generator has been used (Novickij et al., 2015). We have redeveloped the generator by doubling the total discharge capacitance to 0.6 µF and thus increased the total accumulated energy of the pulse to 1.2 J. The RC snubber and crowbar circuits have been adjusted to match the new pulse forming circuit for effective compensation of the reverse transient voltage. The inductor, which served as a load of the system consisted of 11 windings and six layers (total of 66 windings) with a resulting total inductance of 9.8 µH. The inner diameter of the inductor was 3 mm to match the 0.1 mL PCR (Polymerase chain reaction) tube (STARLAB International GmbH, Hamburg, Germany). In this work we applied bursts of 50 pulses at low frequency (1 Hz). The duration of one pulse was 15 µs and maximum amplitude was 5.5 T. There were 50 pulses in one burst, and 2 or 4 bursts were applied. The total pulse number was either 100 (protocol 50 × 2) or 200 (protocol 50 × 4). Between bursts, a 30-second delay was introduced to allow cooling of the sample. The waveform of the pulse was measured by a calibrated B-dot sensor (VGTU, Vilnius, Lithuania) and is shown in Fig. 1. The maximum dB/dt of the pulse was 1.2 × 10^6^ T/s for the rising front and 4.6 × 10^5^ T/s for the falling one.

For conventional electroporation, which served as positive control, the 0–3 kV square wave electroporator (Novickij et al., 2016a) or commercially available Betatech electroporator (Electro cell B10; Betatech, France) have been used. The pulses were generated in a commercially available electroporation cuvette with 1 mm gap between the electrodes (Cuvette Nr. 610; BTX, San Diego, CA, USA or PeqLab, Erlangen, Germany). A single 100 µs square wave pulse of 1.2 kV/cm voltage-to-distance ratio was used in the study.

### Induced electric field

The induced electric field is proportional to the dB/dt of the pulse and is one of the main influencing parameters during PEMF permeabilization. We have evaluated the distribution of the induced electric field in the PCR tube using finite element method (FEM) in Comsol Multiphysics 5.0 software (COMSOL, Stockholm, Sweden). The model of the inductor has been introduced, and the waveform presented in Fig. 1 has been used as a terminal input.

**Figure 2:**
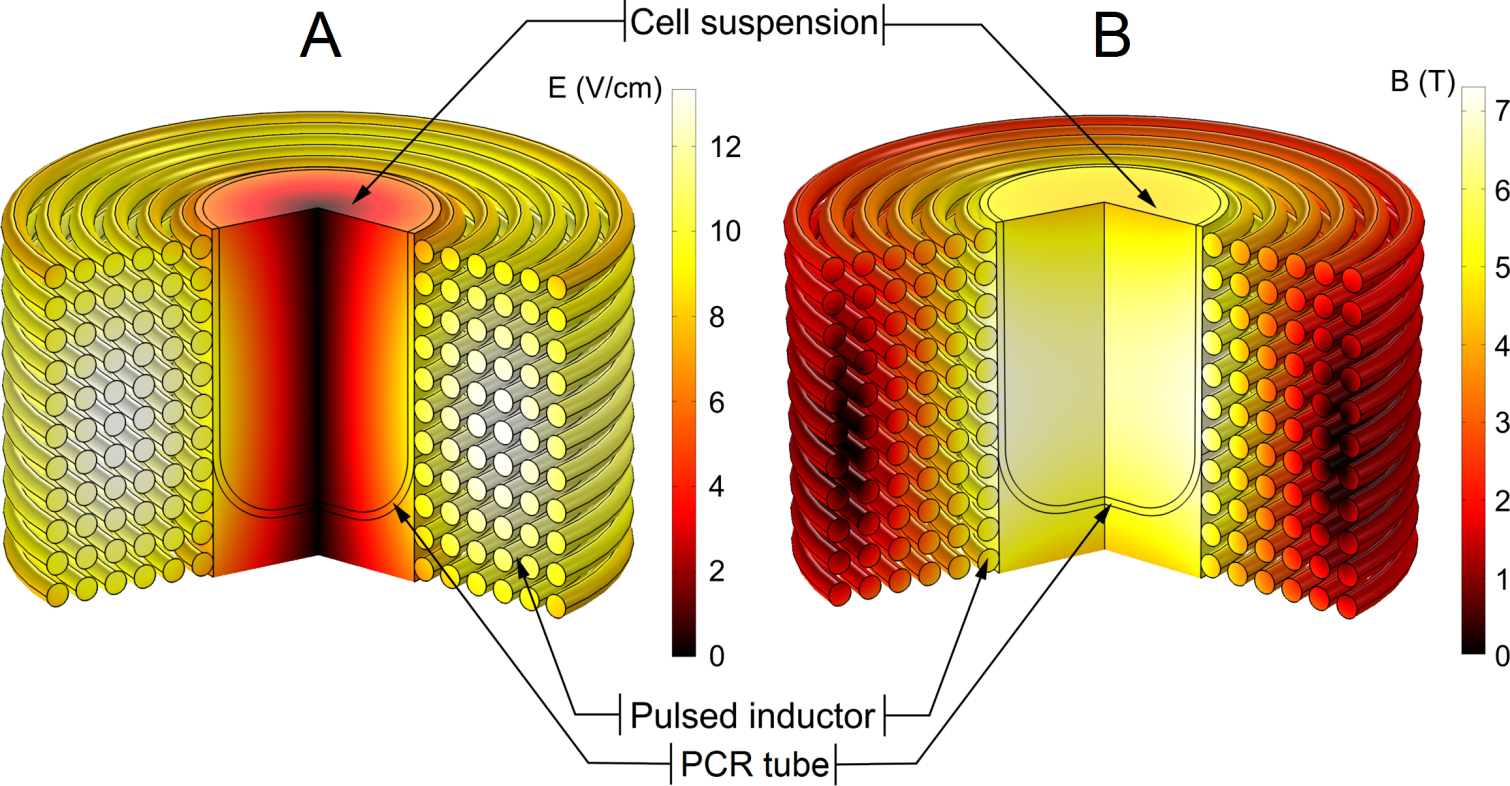
Finite-element method (FEM) model of the pulsed magnetic field inductor. (A) Spatial distribution of the electric field at highest dB/dt of the magnetic pulse; (B) spatial distribution of the magnetic field. The electric field was the highest at the edges of the polymerase-chain-reaction (PCR) tube and decreased to the zero in the centre of the sample while the magnetic field was approximately homogeneous through the whole sample.

The resultant spatial distributions of the magnetic and the induced electric fields are shown in Fig. 2.

The spatial distribution of the induced electric field is highly non-uniform with maximum amplitude near the walls of the inductor (7–8 V/cm) and a linear decrease of the electric field strength towards the centre to 0 V/cm. On the contrary, the spatial distribution of the magnetic field in the effective volume of the inductor is almost uniform (5.5 T ± 10%).

### Cell preparation

Chinese Hamster Ovary cells (European Collection of Authenticated Cell Cultures ECACC, cells CHO-K1, cat. no. 85051005, obtained directly from the repository) were grown in 25 cm^2^ culture flasks (TPP, Trasadingen, Switzerland) in HAM F-12 growth medium (PAA, Kremplstraße, Austria) for 2–3 days in an incubator (Kambič, Semič, Slovenia) at 37 °C and humidified 5% CO_2_. The growth medium (used in this composition through all experiments) was supplemented with 10% fetal bovine serum (Sigma-Aldrich, Darmstadt, Germany), L-glutamine (StemCell, Vancouver, CAN) and antibiotics penicillin/streptomycin (PAA) and gentamycin (Sigma-Aldrich, Germany). On the day of the experiments, the cell suspension was prepared. Cells were detached by 10× trypsin-EDTA (PAA), diluted 1:9 in Hank’s basal salt solution (StemCell) and the trypsin was inactivated by 2.5 ml of the HAM F-12 growth medium. Cells were transferred to a 50 ml centrifuge tube (TPP) and centrifuged 5 min at 180 g and 22 °C. The supernatant was removed, and cells were re-suspended in the growth medium HAM F-12 at cell density 10^7^ cells/ml.

### Cell permeabilization

Right before pulse application, the cell suspension was mixed with fluorescent dyes—either propidium iodide (PI) or YO-PRO^®^-1 (YP). The final concentration of PI was 136 µM, and of YP it was 1 µM (both Life Technologies, Carlsbad, CA, USA). 20 µl of the cells-dye mixture was transferred into a 100 µl PCR tube, followed by the PEMF treatment. The maximal treated volume (20 µl) was limited by the effective volume of the inductor.

Alternatively, for estimation of the number of non-reversibly permeabilized cells or short-term cell death, the cells were PEMF treated without fluorescent dyes. After pulse application, they were transferred to a microcentrifuge tube, 200 µl of the growth medium was added, and the sample was left in an incubator at 37 °C and humidified 5% CO_2_. After 30 min, the PI was added in the final concentration of 136 µM and after 3 min, flow cytometric analysis was performed.

### Fluorescence microscopy

Three minutes after the pulse application, cells were transferred from the PCR tube to a well on a 24-well plate (TPP) and diluted with 1 ml of the phosphate-potassium buffer (KPB) to stop the influx of the PI or YP. The composition of the KPB buffer was 10 mM KH_2_PO_4_/K_2_HPO_4_ in ratio 40.5:9.5, 1 mM MgCl_2_, 250 mM sucrose. MgCl_2_, K_2_HPO_4_, and sucrose were from Sigma-Aldrich, Germany, and KH_2_PO_4_ from Merck, Germany. After 3–5 min the cells settled to the bottom of the dish. Bright-field and fluorescent images were acquired. We used the inverted microscope AxioVert 200 (Zeiss, Oberkochen, Germany) and 20× objective. Samples were excited with a monochromator (High-Speed Polychromator; Visitron Systems GmbH, Puchheim, Germany) at wavelength 490 nm (PI) and 491 nm (YP), and the emitted fluorescence was detected through a 605/55 nm (PI) or 535/30 nm (YP) bandpass filters (models 71006 and 41028; Chroma, Rockingham, USA). Images were acquired using the VisiCam 1280 CCD camera (Visitron, Puchheim, Germany) and the MetaMorph PC software (Molecular Devices, Sunnyvale, CA, USA). For each unique set of parameters, three repetitions were done. The acquired images were analysed in software (ImageJ; National Institutes of Health, Bethesda, MD, USA). First, the background was subtracted, and a fixed value threshold was set for all the images. The number of fluorescent and all cells was manually counted on each image. The percentage of fluorescent cells was calculated as the number of the fluorescent cells normalised to the total number of cells on one image. The generality of the observation has been evaluated based on 7–15 digital fluorescent microscopy images (100+ cells) for each set of parameters. Statistical analysis has been performed in OriginPro 8.5 software (OriginLab, Northhampton, MA, USA). The paired *t*-test was used for evaluation of the statistical significance. The results were considered statistically significant at *P* < 0.05.

### Flow cytometry

Three minutes after the last pulse, the cell suspension was mixed with 180 µl of KPB and transferred to a well on the 96-well plate (PI) or was mixed with 100 µl of KPB and transferred to a 5 ml tube (Sarsted, Nümbrecht, Germany). The 96-well plate was transferred into the Attune Autosampler connected to the flow cytometer (Attune NxT; Life Technologies, Carlsbad, CA, USA) and samples in 5 ml tubes were transferred to a tube holder on the flow cytometer. Cells were excited with a blue laser at 488 nm, and the emitted fluorescence was detected through a 574/26 nm band-pass filter (PI) or 530/30 nm (YP). The measurement was finished when 10,000 (PI) or 30,000 events (YP) were acquired. Obtained data was analysed using the Attune NxT software. On the dot plot of forward-scatter versus side scatter, cells were separated from all events by gating. Fluorescence was determined as the median value of the gated cells of the measured signal (MFI). The fluorescence of each parameter (100 pulses, 200 pulses) was normalised to the control sample. The average and the standard deviation were calculated. The experiments were performed in triplicates in random order.

### Cell survival evaluation

After the pulse application, 10 µl of the cell suspension was immediately transferred to a 1.5 ml microcentrifuge tube, 390 µl of the growth medium HAM F-12 was added, and the cell suspension was mixed using a pipette. Then, cells were plated in 100 µl per well in a 96-well plate (TPP) in three technical repetitions. The plate was transferred to the incubator at humidified 5% CO_2_ and 37 °C for 24 h. Cell survival was assessed via metabolic activity MTS assay (CellTiter 96^®^ AQueous One Solution Cell Proliferation Assay (MTS); Promega, Madison, WI, USA). The MTS tetrazolium compound is reduced by living cells into a colored formazan product, and the number of metabolically active cells is proportional to the measured absorbance. 20 µl of the MTS assay was added per well and after 2 h the absorbance at 490 nm was measured with a spectrofluorometer (Tecan Infinite 200; Tecan, Grödig, Austria). The survival was calculated by normalizing the average absorbance of the three technical repetitions of the samples to the absorbance of the control.

### Thermal effect evaluation

The temperature rise due to Joule heating was measured by the fiber optic sensor system (opSens, Québec, CAN), which consisted of ProSens signal conditioner and a fiber optic temperature sensor OTG-M170. The sensor has been put inside the PCR tube filled with the cell suspension, and a burst of 50 pulses of maximum amplitude has been generated. The inductor has been cooled with ice during the pulsing to minimize the temperature rise.

## Results

The CHO cells have been subjected to bursts (50 × 2 and 50 × 4) i.e., 100 and 200 pulses of 5.5 T at pulse repetition frequency of 1 Hz, first followed by fluorescence microscopy analysis.

**Figure 3:**
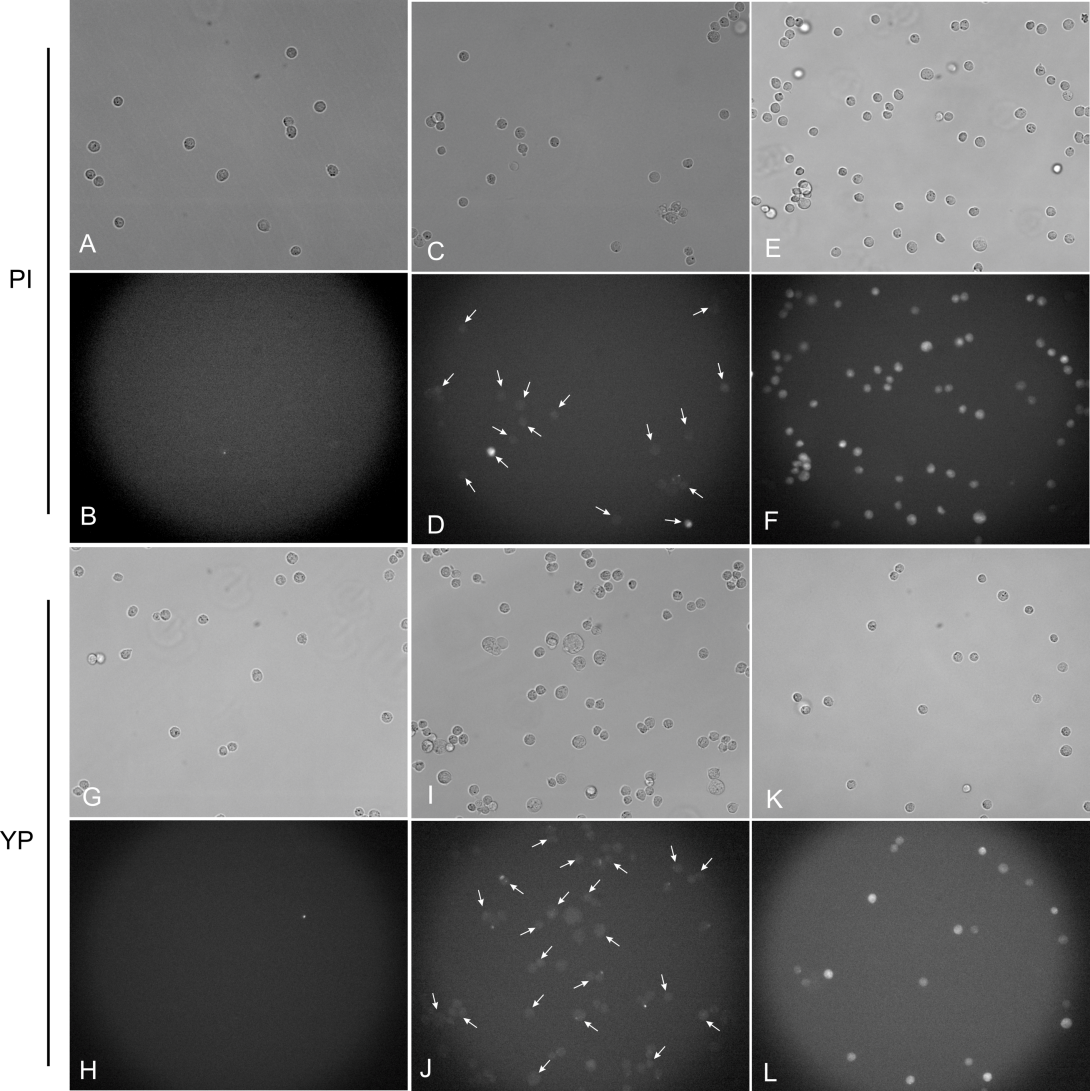
Light and fluorescence microscopy images using PI and YP fluorescent dye assays. (A, B, G, H) untreated control; (C, D, I, J) cells after the 50 × 4, 5.5 T pulsing protocol; (E, F, K, L) cells after the single 100 µs, 1.2 kV/cm pulsing protocol. Arrows are used to highlight the cells with weak mean fluorescence, which were counted as PI and YP positive during analysis.

The fluorescence microscopy images, which accurately represent the generality of the observation after the PEMF and PEF induced permeabilization, are shown in Fig. 3. First, the PI assay was used. The untreated control sample showed no detectable PI fluorescence and thus cell permeabilization (Figs. 3A and 3B). Figures 3C and 3D show the cells exposed to 50 × 4, 5.5 T PEMF treatment and the majority of the cells being PI positive. However, the fluorescence intensity of permeabilized cells was weak. As a positive control, the PEF pulse (1 × 1.2 kV/cm 100 µs) was introduced (Figs. 3E and 3F), resulting in high permeabilization of CHO cells. If compared to PEMF treatment the PEF induced higher uptake of PI (Figs. 3D and 3F).

Secondly, the YP assay was used. The untreated control sample (Fig. 3G and 3H) again showed no permeabilization. Further, we were able to detect YP uptake when the 50 × 4, 5.5 T PEMF treatment was applied (Figs. 3I and 3J). Also, the fluorescence of cells was more definitive compared to the PI assay (Fig. 3D). Lastly, the positive PEF control (Figs. 3K and 3L) showed higher uptake of YP (compared to PEMF treatment, Fig. 3J) and the majority of cells being permeabilized, which is in agreement with the result when PI was used with the positive control (Fig. 3F).

**Figure 4:**
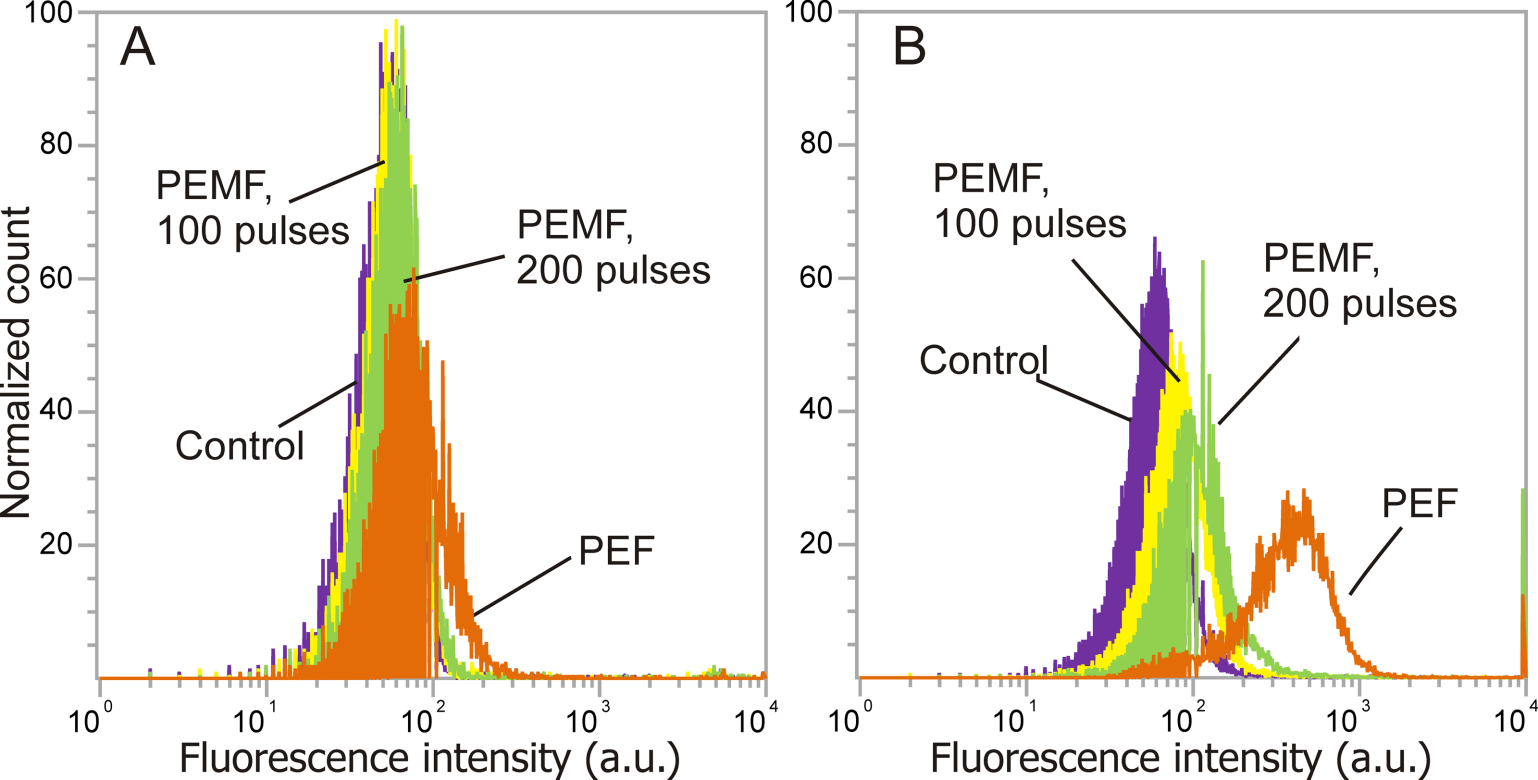
The dependence of fluorescence intensity spectra (PI, YP) on the treatment parameters. After pulsed treatment a shift of fluorescence spectra was detected indicative of membrane permeabilization, where (A) PI assay; (B) YP assay; violet, untreated control; yellow, 50 × 2, 5.5 T PEMF; green, 50 ×4, 5.5 T PEMF; orange, PEF (1 × 100 µs, 1.2 kV/cm).

Further, the flow cytometric analysis was performed. The characteristic shifts of fluorescence spectra due to applied PEMF or PEF treatment are shown in Fig. 4. It can be seen that YP fluorescence intensity was higher if compared to the PI assay, which is in agreement with the microscopy data. As a result, both PEMF and PEF treatments showed definitive shifts of fluorescence spectra indicating uptake of YP by the CHO cells. The PI assays showed poor sensitivity and the difference between the untreated sample and PEF or PEMF treatment was less distinguishable.

**Figure 5:**
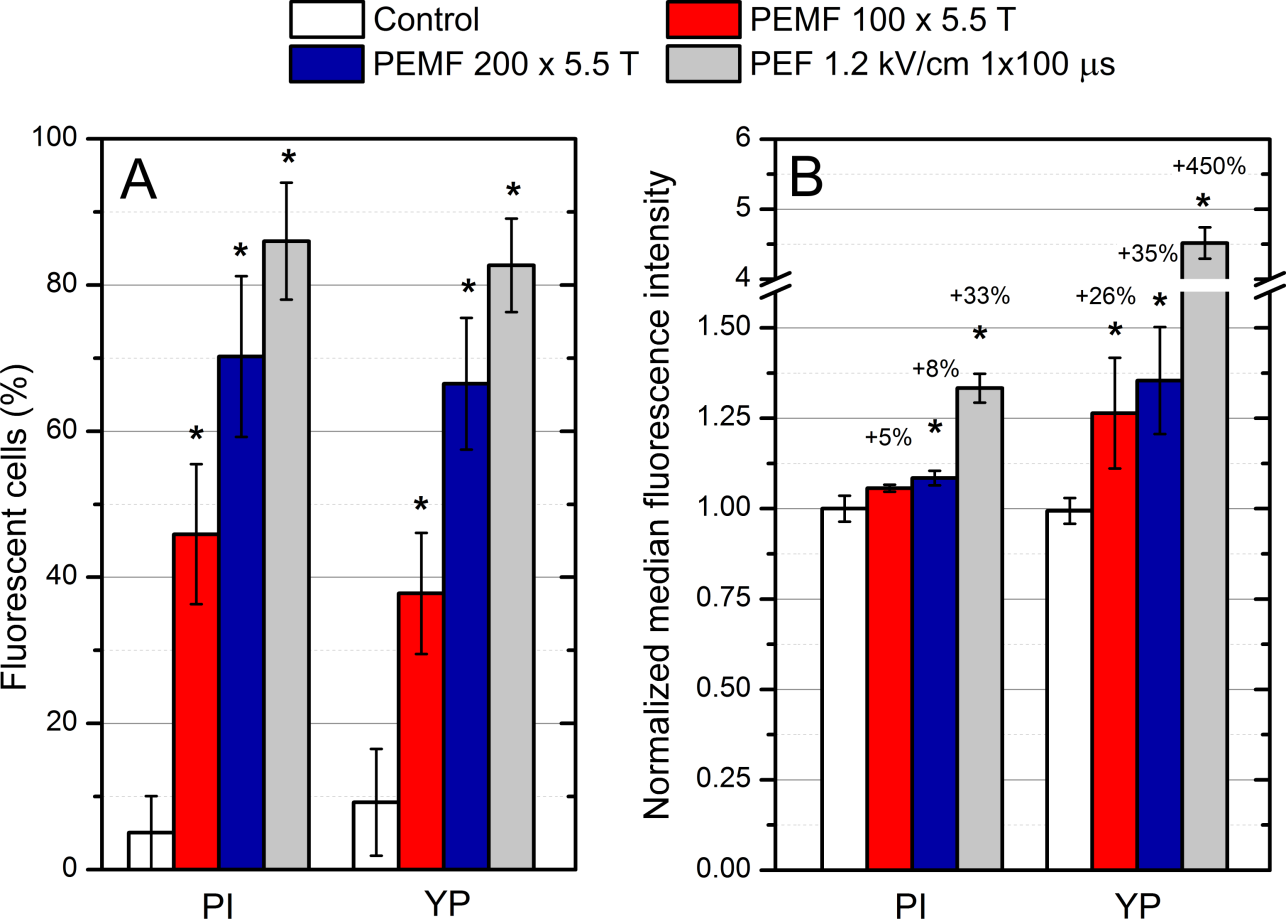
Membrane permeabilization of mammalian cells using bursts of high magnetic field pulses. The percentage of permeabilized cells (A), as well as the median fluorescence (B) increased with the treatment intensity using both PI and YP assays. Asterisk (*) highlights statistically significant difference versus control (*P* < 0.05).

The summary of data on the increase of PEMF and PEF induced cell permeabilization is presented in Fig. 5. Figure 5A shows the percentage of fluorescent cells as determined by fluorescent microscopy and Fig. 5B shows the normalized median fluorescence as determined by the flow cytometry both as a function of different treatment parameters for both PI and YP.

The number of PI-positive cells detected was 70 ± 11% of all cells, when the 50 × 4, (i.e., 200) treatment was used. The result is similar also for the YP assay, which showed 67 ± 9% permeabilization. The 50 × 2, (i.e., 100) protocol was inferior to the 200 PEMF pulses treatment, resulting in 46 ± 10% and 38 ± 8% permeabilization for PI and YP assay, respectively. The fraction of permeabilized cells after PEF pulse (100 µs 1.2 kV/cm), i.e., the positive control, was 86 ± 8% for PI and 83 ± 6% for YP. All of the applied pulsed protocols (PEMF, PEF) showed a statistically significant difference in the number of permeabilized cells if compared to control, independently from the used fluorescent dye.

Further, the data on the increase in median fluorescence intensity (MFI) acquired by flow-cytometric analysis was compared. As it can be observed in Fig. 5B in both cases (PI, YP) the PEMF treatment increased the fluorescence intensity of cells in a dose-dependent manner, which is in agreement with the fluorescence microscopy data. The PI assay was less sensitive for detection of increased molecular uptake compared to YP (based on flow cytometry). For example, the 50 × 4, 5.5 T PEMF treatment induced only 8% increase for the PI while for YP, 35% increase of median cell fluorescence was observed (normalized to control). The PEF (single pulse 100 µs 1.2 kV/cm) treatment showed a 33% increase in fluorescence for the PI, while for the YP it was much higher (450% increase).

**Figure 6:**
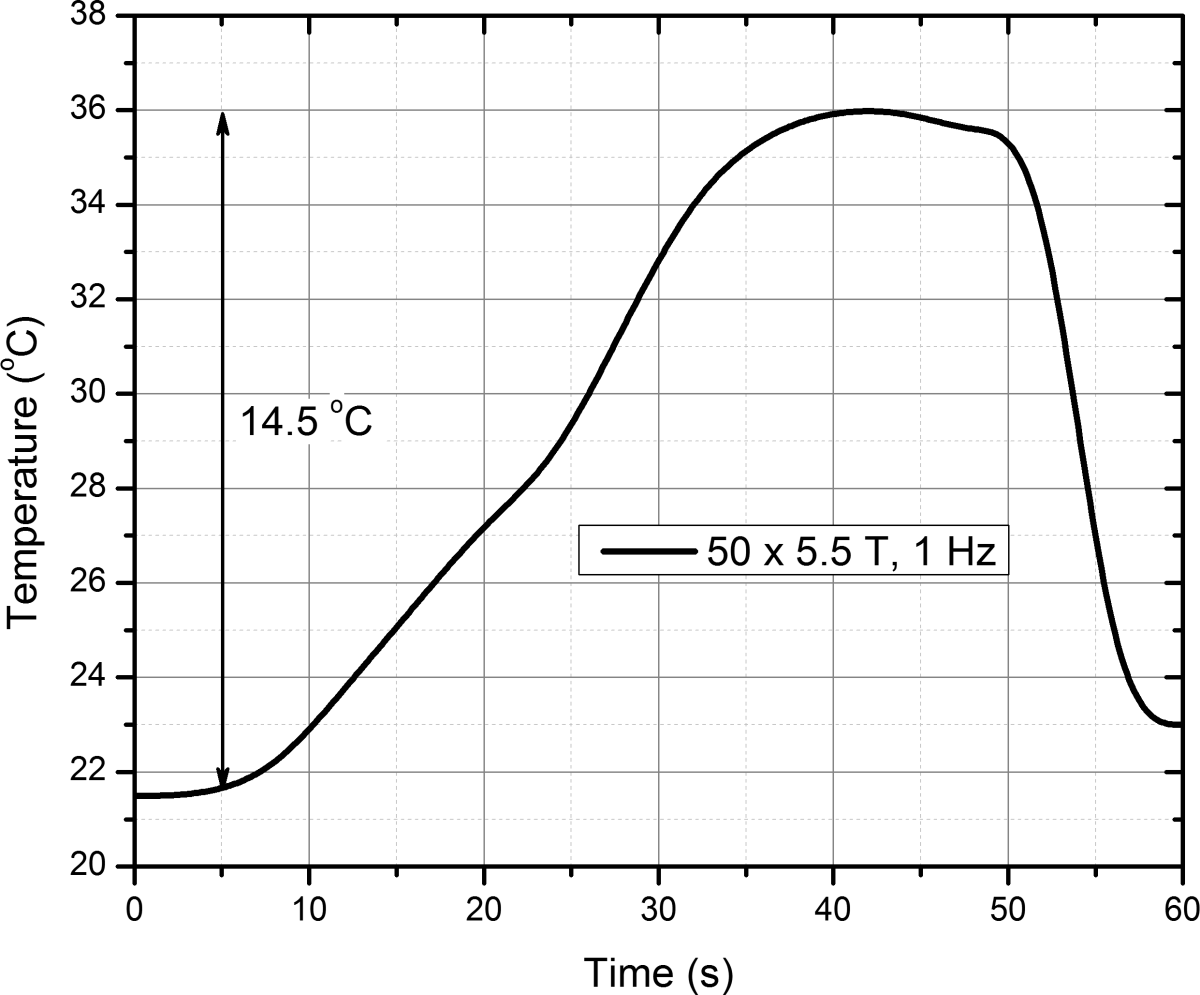
Temperature rise in the cell suspension during PEMF treatment. The temperature rise during PEMF treatment (5.5 T, 50 pulses) did not exceed 14.5°reaching 36 °C and followed by a rapid decline (due to cooling) when the pulsing stops.

We have also evaluated the possible influence of the Joule heating on the treatment outcome. The 50 pulses delivered as a burst result in a maximum temperature rise of 14.5 °C, reaching 36 °C, followed by a rapid decline (due to cooling) when the pulsing stops (See Fig. 6). For the inductor to cool down to room temperature (21−22 °C), a 30 s delay between the bursts (50 pulses) has been made. Two protocols of 50 × 2 (total treatment time of 2 min 10 s) and 50 × 4 (total treatment time of 4 min 50 s) with a maximum amplitude of 5.5 T have been evaluated. It has been experimentally confirmed that the temperature does not exceed 37 °C in both cases. Subsequently, the influence of the temperature on the CHO cells’ permeability has been tested by incubating cells for 5 min using a water bath at 37 °C with PI or YP and measuring the fluorescence by flow cytometry. The PI assay showed an average 5 ± 2% increase of MFI if compared to control, while YP assay did not show any detectable difference in fluorescence (data not shown). Also, neither PEMF nor PEF protocols that were applied in the study resulted in a detectable effect on cell viability (MTS). The PI assay 30 min after the PEMF treatment measured by flow cytometry also showed no increase in fluorescence that would indicate a decrease in short-term cell survival for both protocols and the control (data not shown).

## Discussion

In our study, we demonstrated that the application of PEMF with a low-frequency high dB/dt microsecond range pulse protocol induces permeabilization of CHO cells *in vitro* while not affecting their survival. We have shown that both the 50 × 2 and 50 × 4, 1 Hz protocols result in significantly higher membrane permeability to two different fluorescent dyes compared to the untreated control. The uptake of fluorescent dyes was detected using two experimental techniques—fluorescent microscopy and flow cytometry. Due to the low measured fluorescence signal (indicating weak permeabilization) we have determined the percentage of the fluorescent cells using microscopy instead of flow cytometry since even a small change in the gate significantly affects the result, and we can unintentionally introduce bias in the results. In microsecond range electroporation experiments, the peak in fluorescence usually shifts for one to two decades (Rols, Teissie & Teissié, 1998) and gating does not substantially affect the results. We believe that determining the median fluorescence by flow cytometry is more objective, as we need only to gate the cells from the debris on the side and forward scatter dot plot. Also, the increase in PI fluorescence was lower than YP fluorescence measured with the flow cytometry. The same trend of lower fluorescence of PI with respect to YP was observed in positive controls. One possible reason is smaller sensitivity of the flow cytometry in the range of propidium fluorescence, which could be a limitation of our available infrastructure. The available bandpass filter (574/26 nm or 695/40 nm) filter out the peak of PI fluorescence (636 nm). As a result, only high permeabilization may appear as statistically significant. The second reason could be that YP is more sensitive and membrane is more permeable to YP since it is slightly smaller (630 Da vs. 668.4 Da) than the PI (Idziorek et al., 1995; Bowman et al., 2010). The insufficient PI sensitivity during weak membrane permeabilization (i.e., nanosecond pulses) has been reported before (Pakhomov et al., 2007), while YP showed higher sensitivity and positive results (Vernier, Sun & Gunderesen, 2006).

Nevertheless, we have determined that the fraction of fluorescent cells, as well as the fluorescence, increased with the increase of the PEMF treatment intensity (i.e., a number of pulses) using both the PI and YP assays. The result is agreement with the results of Towhidi et al. (2012) and Kardos & Rabussay (2012). At the same time, the percentage of permeabilized cells after the PEMF treatment was relatively high (up to 70%), but the fluorescence intensity was low. The uptake achieved using conventional PEF treatment was much higher than with the PEMF treatment. The reason could be the low induced electric field during PEMF (7–8 V/cm), however we used higher number of pulses (up to 200 for PEMF) to compensate the difference in intensity. Such a strategy (increasing the number of pulses) is in agreement with the literature. The proof of concept that it is possible to electrotransfer DNA to *E. coli* using 50–150 V/cm 0.1–10 Hz using alternating electric field or induce cell concentration fluctuations was already shown (Xie & Tsong, 1990; Dimitrov, Stoimenova & Tsoneva, 2002). Nevertheless, in our work, the difference in pulse shapes between the applied two methodologies complicate the straightforward parametrical comparison. Additionally, we have a high pulsed magnetic field component (3.3 T), which may have an impact on permeabilization, thus it is plausible that we are observing a superposition of different effects, consequence of several mechanisms working together (Novickij et al., 2016b).

There were several possible mechanisms suggested in the literature—electro-endocytosis (Towhidi et al., 2012; Shankayi et al., 2014; Kranjc et al., 2016), electrophoresis (Towhidi et al., 2012), creation of metastable membrane pores via interaction with membrane-attached magnetic particles (Towhidi et al., 2012), electroporation due to induced electric field (Novickij et al., 2014; Kranjc et al., 2016), changed receptor binding or activation (Shankayi et al., 2014), and induction of magnetomotive force which interacts with ions (Kardos & Rabussay, 2012; Novickij et al., 2016b). In addition to above-mentioned mechanisms, lipid peroxidation could also be a possible mechanism, when the oxidation of the membrane components results in increased susceptibility of the cells to the pulsed electric field (Vernier et al., 2009; Runas & Malmstadt, 2015).

Nevertheless, it is currently still not possible to determine mechanisms and pathways of increased molecular transmembrane transport induced by the PEMF. However, taking into account the acquired data it can be concluded that the PEMF induced contactless permeabilization depends on the pulse parameters in a dose-dependent manner. Also, similarly to electroporation, the effect depends on the electric field amplitude (in this case, an induced electric field), we were able to observe higher cell permeabilization compared to previous works with lower dB/dt exposure (Novickij et al., 2015). Further research is required to provide a better understanding of the process. The limitations of the PEMF methodology should be also addressed in the future i.e., the management of thermal influence during the PEMF treatment, small volume of effect and non-homogeneity of induced electric field. The thermal effects during proposed methodology are influenced mainly by the Joule heating in the inductor itself due to high current (>500 A), while the influence of eddy currents due to high dB/dt treatment is negligible. Without additional ferromagnetic nanoparticles, which are used in PEMF induced hyperthermia works, the effect is limited to Ohmic heating in the suspension (Gaitas & Kim, 2015). In our case, the short exposure time, low repetition frequency (enough time for convective heat transfer) and limited amount of pulses, all diminish the effect of eddy currents. Therefore, successful management of the Joule heating will allow further increase of the energy and number of PEMF pulses and thus introduce higher flexibility for parametric analysis. Finally, the solutions for increase of the volume of effect and improvement of the non-homogeneity of induced electric field during PEMF treatment should be provided, which is a matter of pulsed magnetic field inductor and generator design. These limitations will be addressed in the scope of future works.

##  Supplemental Information

10.7717/peerj.3267/supp-1 Supplemental Information 1 The link and the explanation of the raw data archive contents

## Additional Information and Declarations

### Data Availability

The following information was supplied regarding data availability: Figshare 10.6084/m9.figshare.4626298.
https://figshare.com/s/0717f5e3dc11667c6785.
