# Supplementary material for: Membrane permeabilization of mammalian cells using bursts of high magnetic field pulses
Source: PeerJ. 2017 Apr 26;5:e3267. doi: 10.7717/peerj.3267 (PMC5408723; doi:10.7717/peerj.3267)
Supplement: Supplemental Information 1 [file peerj-05-3267-s001.pdf]

The link to repository was provided: https://figshare.com/s/0717f5e3dc11667c6785

The archive includes raw data:

- Measured pulse waveforms
- PI and YP microscopy images
- Flow cytometry raw data
- COMSOL model of the inductor

Each set of data is provided in a separate folder, where the file names have been chosen to comply with the parameters of treatment that was used in the study.
